# Trends in changes of family functioning during different phases of the pandemic – findings across four population-based surveys between 2020 to 2023 in Germany

**DOI:** 10.1186/s12889-024-20650-2

**Published:** 2024-11-20

**Authors:** Alina Geprägs, David Bürgin, Jörg M. Fegert, Elmar Brähler, Vera Clemens

**Affiliations:** 1 Hospital of Child and Adolescent Psychiatry/Psychotherapy, University of Ulm, Ulm, Germany; 2 Child and Adolescent Psychiatric Research Department (UPKKJ), Psychiatric University Hospitals, University of Basel, Basel, Switzerland; 3 Jacobs Center for Productive Youth Development, University of Zurich, Zurich, Switzerland; 4 Department for Psychosomatic Medicine and Psychotherapy, University Medical Center of Johannes Gutenberg University of Mainz, Mainz, Germany; 5 Integrated Research and Treatment Center Adiposity Diseases, Behavioral Medicine Unit, Department of Psychosomatic Medicine and Psychotherapy, Leipzig University Medical Center, Leipzig, Germany

**Keywords:** COVID-19 pandemic, Family functioning, Quality of life, Relationship with child and partner, Mental health

## Abstract

**Background:**

The COVID-19 pandemic and the associated measures have had a significant impact on millions of individuals and families worldwide. Although cross-sectional studies have demonstrated the considerable burden placed on families during the pandemic, trends over different phases of the pandemic including later stages and using population-based samples is scarce.

**Objective:**

In this study, we aimed to assess trends in family functioning across four population-based surveys between December 2020 and March 2023 using a repeated cross-sectional design. The surveys were conducted using a similar sampling strategy and measures. We included individuals residing in a household with at least one minor below the age of 16.

**Results:**

The most notable changes across surveys over time were related to quality of life. While 54.3% of respondents reported a decline in quality of life during the winter of 20/21 compared to pre-pandemic levels, this was observed in only 22.6% of participants during the spring of 23. The proportion of respondents who indicated a deterioration in their relations with their children also decreased during the pandemic. While 9.9% of respondents reported a deterioration in their relationship with their children during the winter of 20/21 in comparison to the initial phase of the pandemic, this was reported by only 5.2% in the spring 23. The relationship with one’s partner and health status exhibited minimal fluctuations. Mental health problems were associated with a decline in quality of life, health status and relationships with children and partners compared to pre-pandemic levels at all time points. Moreover, lower income levels were associated with poorer relationship quality with the partner in the most recent wave.

**Conclusions:**

Our findings demonstrate significant improvements in family functioning since the onset of the pandemic, indicating that individuals and families in our sample were generally adapting well. However, a subgroup of the population still reports suboptimal family functioning compared to before the pandemic. Psychosocial care and social policy support for families are needed.

**Supplementary Information:**

The online version contains supplementary material available at 10.1186/s12889-024-20650-2.

## Introduction

In late 2019, the SARS-CoV-2 virus, which causes the disease known as “coronavirus disease 2019” (abbreviated as “COVID-19”), spread rapidly from its point of origin in Wuhan, China, to other parts of the world. The death rate associated with this virus was approximately 20% at the beginning of the outbreak and claimed many fatalities [1]. Furthermore, the rapidly growing cases posed a significant risk of overwhelming the nation’s healthcare infrastructure. In response to this threat to healthcare systems, governments implemented different measures, including contact restrictions and curfews, the closure of schools, childcare institutions, leisure time activities and cultural and gastronomic venues [2]. These measures were necessary and effective in preventing the overload of the health care system resources [3, 4].,In Germany, for example, they have resulted in relatively low mortality rates in compared to other countries (Konishi, 2024), thereby saving lives. Nevertheless, research has demonstrated a range of adverse effects on mental health and quality of life [5–7]. These included an increase in depressive symptoms since the beginning of the COVID-19 pandemic, confirmed by a meta-analysis conducted by Ludwig-Walz et al. (2022) [7]. This effect was further amplified by the implementation of additional restrictive measures and school closures, potentially due to a lack of access to mental health support [8]. In a similar way, anxiety was also found to be affected [9]. Thus, we see the enormous psychosocial cost of these measures to the population, despite their undisputed efficacy and necessity.

However, some measures had a differential impact on specific subgroups of the population. The closure of schools and childcare institutions, for instance, had a significant impact on families, especially those with young children. For a long time during the pandemic, the effects of restrictive measures on children and their mental health, like a lack of contact with peers or a reduction in stress regulation capabilities, received less attention in public and governance discussions than they deserved [10]. Fegert et al. warned in a very early stage of the pandemic of these lasting negative consequences of the pandemic and the restrictive measures, especially for children [11]. However, it was not only the children who were strongly impacted by events such as school closures. In general, families with minor children reported specific stressors, including the challenge of balancing childcare and work duties [12] and the disruption of daily routines and heightened uncertainty [13]. As key factors influencing these aspects of family functioning, the quality of relationships with the partner and children should be considered [14]. During the pandemic and with increasing stress, the parent-child relationship appeared to be adversely affected [15]. A total of 4% of parents indicated that relationship difficulties constituted a stressor during the pandemic [16]. A greater proportion of caregivers reported that the child-parent relationship was less close and that there were more conflicts than before the pandemic [17]. Conversely, a positive marital quality has been identified as a protective factor for mothers against depression during the pandemic [18]. Additionally, elevated levels of parental stress and burnout have been reported [19, 20]. This heightened parental stress was associated with a range of subsequent outcomes, including an increased risk of child abuse [16] and heightened behavioural problems of children [21]. Concerning child maltreatment, the findings are highly heterogeneous [22, 23], due to the different forms of investigation employed. Some studies indicate an increase during the pandemic (e.g [24]), while others suggest a decrease [25]. Studies interrogating families indicate an increase in the use of physical punishment, including spanking or hitting children, by 2–20% [26, 27]. In sum, research suggests that the pandemic has resulted in an increase in parent-child conflicts, as well as a rise in parental conflicts and parental stress. These findings, when viewed in conjunction with various stressors, underscore the high burden that the pandemic has placed on families. This highlights the necessity for future research to prioritize the examination of family functioning.

Importantly, not all individuals have been impacted by the aforementioned adverse effects on the parent-child relationship in the same manner. As protective factors for the parent-child relationship, the female gender of the child, school factors, parental education and stricter lockdown measures were identified [28]. Associations between elevated depressive symptoms among caregivers and an increased prevalence of child-parent relationship conflicts have been identified [24]. Furthermore higher perceived child stress was linked to elevated child-parent relationship conflicts and diminished levels of closeness in the child-parent relationship [17]. Several risk groups have been identified as being particularly vulnerable to the negative consequences of the pandemic. For instance, female gender of adults was associated with a reduced quality of life [19, 29], while financial strain was linked to a reduced quality of life [30, 31]. This includes having mental health problems [30] and parental stress [32]. Together, several sociodemographic and psychological variables emerged as important predictors of pandemic-associated changes in different outcomes and should therefore be included as potential covariates.

Over the course of the three-year period of the COVID-19 pandemic, infection cases fluctuated perspicuously. Additionally, the advent of the vaccine altered the dynamic of the pandemic. Consequently, the pandemic was characterized by different stages, each accompanied by a unique set of implemented measures to flatten the infection curve. For the first months of the pandemic longitudinal research was slightly heterogeneous. Some studies have indicated that the initial burden was high in April 2020, with a subsequent slight decrease [33, 34]. In addition, a meta-analysis has described a peak in the mental health burden for April and May 2020 [35]. Other studies showed a decrease in anxiety and depression from March to June 2020 [7] and a decline in October 2020 compared to May 2020 [36]. Concerning children and adolescents, a representative study indicated a decline in mental health and quality of life since the beginning of the pandemic [37]. This decline remitted in part to still higher levels within pre-endemic measures compared with those prior to the pandemic [37].

Taken together, longitudinal research conducted during the pandemic has primarily focused on mental health and quality of life. Other important aspects of family functioning and individual variables, such as the quality of relationships and the health status have been largely overlooked in research. These factors warrant consideration for the development of intervention and prevention strategies in the context of future crises. Besides, the majority of longitudinal research compared results from pre-pandemic data with several early stages of the pandemic [34, 38–48]. This provides profound insights into changes occurring during the early stages of the pandemic: However; it fails to capture trends in changes occurring until the endemic phase in 2023. In addition, some studies focused only on specific groups like children and adolescents [41, 42, 44] or healthcare professionals [49]. Within this study, we aimed to investigate family functioning during different stages of the COVID-19 pandemic with a repeated cross-sectional design, collecting data from four German population-based surveys conducted between 2020 and 2023, hypothesizing an decrease in family functioning during the pandemic and an improvement after the end of the pandemic. First, we investigated changes in the relationship quality between parents and between parents and their children, as well as changes in the individual quality of life and health status of the target parent. Second, we examined the association between several risk factors (low income, symptoms of depression and anxiety, number of children and gender) and family functioning, as we described above the potential association to these covariates. To the best of our knowledge, this is the first study to investigate family relationships across different stages of the pandemic including later stages up to 2023, using large representative samples recruited with similar methodology in Germany.

## Methods

### Sample procedure

We used four independent cross-sectional probability samples, representative of the German population. All four samples were obtained by a demographic consulting company (Unabhängiger Service für Umfragen, Methoden und Analysen (USUMA), Berlin, Germany), using the same sample procedure as described in the following. Arbeitskreis Markt- und Sozialforschungsinstitute e.V. (ADM) systematic area sampling was employed. This covers the entire inhabited area of Germany and is based on the municipal classification of the Federal Republic of Germany. Therefore, Germany was subdivided in multiple steps to ensure that the distribution of private households in Germany was proportionately represented. Next, using a random route procedure, private households were selected systematically. In the event that more than one individual within a given household met the established study criteria, a single individual was randomly selected from the household using a Kish-selection Grid technique. To be included in the study, participants had to be at least 16 years old and have sufficient German language skills (assessed by the research staff during the introduction and explanation part). In a first step, the selected individual was informed about the procedure and the research background and provided informed consent. In a second step, a face-to-face interview was conducted to gather information on basic sociodemographic characteristics. This took place at the residence of the participant. Afterwards, the main part of the questionnaire was completed by the participants alone with a research associate staying in the same room in order to be able to help in case of uncertainties and questions, also regarding the language, with a researcher present to address any queries that may arise. Next, the data was combined in a database devoid of identifying information. Throughout the course of the interviews, the researchers adhered to the established hygiene protocols, including the use of masks, maintaining social distancing and hand hygiene. All four studies were conducted in accordance with the Declaration of Helsinki and were approved by the Ethics Committee of the Medical Department of the University of Leipzig. The first study was conducted during the second wave of COVID-19 in Germany from December 14th, 2020 to March 7th, 2021. During this time, a series of measures were implemented, including the closure of educational institutions, the suspension of commercial activities and the imposition of stringent social distancing protocols. In detail, schools and childcare facilities were only accessible for emergency purposes, while essential services such as supermarkets and pharmacies remained open. Social gatherings were permitted with only one individual from another household. The second study took place prior to and during the initial phase of the fourth wave of COVID-19 in Germany, between July 28th and October 1st, 2021. During this period, shops and school or childcare facilities have resumed operations in Germany. Private meetings were permitted with a maximum of 25 individuals, depending on the local infection rates. The obligation to wear masks was implemented for use within buildings. The third study was conducted between March 3rd and May 26th, 2022. In March, the remaining restrictions in Germany were lifted, with the exception of the obligation to wear masks at certain places like public transportation. The fourth study took place between December 12th, 2022 and March 17th, 2023. During this period in Germany, there were no lockdown measures anymore and even the obligation to wear masks in certain places was lifted. Concerning the combined sample of the four studies, only three or fewer participants identified as a “diverse” gender andwere therefore excluded due to the relatively small number. The response rate for all studies was approximately 40%. For detailed information regarding the reasons for non-participation see supplement Tables 2, 3, 4 and 5. For the purposes of this analysis, we combined subsamples in a single database. For each time point, data were only included from participants with children under the age of 16 in their household. Therefore, the first study was based on a subsample of *n* = 438, the second on a subsample of *n* = 453, the third on a subsample of *n* = 499 and the fourth on a subsample of *n* = 439. Overall, the total sample was *N* = 1,829.

### Measures

*Symptoms of depression and anxiety* were assessed using the Patient Health Questionnaire 4 (PHQ-4) [50]; German version [51]. The PHQ-4 consists of four items, comprising two items each for assessing anxiety and depression. The participants were divided into two groups based on the presence or absence of symptoms of depression and anxiety. The first group, designated as “no symptoms of depression and anxiety,” consisted of values from 0 to 3, while the second group, labeled “with symptoms of depression and anxiety,” included values from 4 to 12. *Change in relationship with partner*, *change in relationship with child*, *change in quality of life and change in health status* were measured using a single-item self-rating question (“Compared to before the COVID-19 pandemic:“ “…how would you describe your current relationship with your partner?”;,“, how would you describe your current relationship with your child(ren)?”, “… how would you describe your current quality of life?”; and “… how would you describe your health status?”). They were presented with a series of options, ranging from 0 (“Much better than before the pandemic”), 1 (“Slightly better than before the pandemic”), 2 (“Equal to before the pandemic”), 3 (“Slightly worse than before the pandemic”) to 4 (“Much worse than before the pandemic”). The answer options were combined and recoded into three categories: 1 (“Worse than before the pandemic”) including the options “Slightly worse than before the pandemic” and “Much worse than before the pandemic”, 2 (“Equal to before the pandemic”) including the option (“Equal to before the pandemic”) and 3 (“Better than before the pandemic”) including the options “Slightly better than before the pandemic” and “Much better than before the pandemic”. The recoded variables were used to assess changes in relationships prior to and throughout the pandemic via the use of confidence intervals. Change in the relationship with the partner was not assessed in spring 23. *Income* was divided into two groups, based on the poverty level in Germany for the equivalent income, published by the Federal Office of Statistics of Germany [52]. The equivalent income was calculated by dividing the household income through the root of the number of individuals living on that income with given values for children. The resulting groups were those with incomes below the poverty level and those with incomes above the poverty level. The *number of children* in each household was assessed by enumerating the number of children under the age of 16 living in the household. The data were then divided into three groups: one child, two children and three or more children.

### Statistical analysis

All analyses were performed using SPSS version 28 and R (version 4.3.1). Only valid data were included in these analyses. In a first step, the changes in the quality of relationships, quality of life, and health status were descriptively presented. In the inferential main analysis, to examine significant differences between time points, 95% confidence intervals were calculated for each percentage. To this end R (version 4.3.1) and the “interpretCI” package with the ”propCI()” command were employed. This command calculates the confidence interval for proportions using sample size, proportion and confidence level. Two-sided confidence intervals were calculated with a 95% confidence level, meaning with 95% confidence the true percentage lies within the confidence interval. Overlapping confidence intervals represent no significant difference between two groups or time points and non-overlapping confidence intervals represent significant differences. In a second step, the variables (changes in the quality of relationships, quality of life, and health status) were explored, stratified by income, gender, number of children, symptoms of depression and anxiety and visualized in a line plot. Furthermore, we calculated binary logistic regression analysis for each dependent variable (change in health status, change in quality of life, change in relationship with child, change in relationship with partner). Therefore, the dependent variables were divided into two groups: the first group containing participants who reported a worsening in the variable compared to before the pandemic and the second group containing participants who reported no change or an improvement in the variable. These binary variables were used as dependent variables in the four models. As independent variables changes between the time points, as well as the covariates income, symptoms of depression and anxiety, gender and number of children were used to include these possible confounders. The changes between the time points were investigated using staircase coding, which divided the time point variable into four manifestations (winter 20/21, summer 21, spring 22, spring 23) within a coding scheme comprising three variables. The first variable represents the differences between winter 20/21 and summer 21, the second variable the differences between summer 21 and spring 22 and the third variable the differences between spring 22 and spring 23 [53] (Walter et al., 1987). Stair case coding was used to divide the number of children variable with three manifestations into two variables. The first variable represents the difference between one and two children and the second variable represents the difference between two and three children.

## Results

The final samples included 438 participants (*N* = 266, 60.70% women) for the sample in winter 20/21 and 415 participants (*N* = 257, 61.90% women) for the sample in summer 21. A total of 499 participants (*N* = 267, 53.50% women) were included in the sample for spring 22 and 439 participants (*N* = 273, 62.20% women) in the sample for spring 23. Means and standard deviations of age were 36.58 (9.13) for winter 20/21, 37.27 (9.81) for summer 21, 36.94 (9.87) for spring 22 and 37.85 (9.66) for spring 23. The characteristics of the sample are presented in Table 1. In the following, the results are described and presented in figures. For detailed confidence intervals, see supplement Tables 6, 7, 8, 9 and 10.

**Table 1:** Sample characteristics for the four samples

	Winter 20/21		Summer 21		Spring 22		Spring 23	
Variable	M	SD	M	SD	M	SD	M	SD
Age	36.58	9.13	37.27	9.81	36.94	9.87	37.85	9.66
	N	%	N	%	N	%	N	%
Female Gender	266	60.70	257	61.90	267	53.50	273	62.20
Income under poverty level	76	18.01	63	15.63	70	14.29	70	14.29
Symptoms of anxiety and depression	63	14.52	56	13.49	68	13.65	66	15.10
Number of children in the household below the age of 16								
One child	241	55.02	242	58.31	291	58.32	287	65.40
Two children	171	39.04	148	35.66	176	35.27	134	30.50
Three or more children	26	5.94	25	6.02	32	6.41	18	4.10

Presented as mean, standard deviation and range for continuous variables and frequency and percentage for categorical variables. Winter 20/21(*N* = 422–438); Summer 21 (*N* = 403–415); Spring 22 (*N* = 490–499); Spring 23 (*N* = 431–439)

### Change in relationship quality, quality of life and health status across different stages of the COVID-19 pandemic

**Fig. 1:**
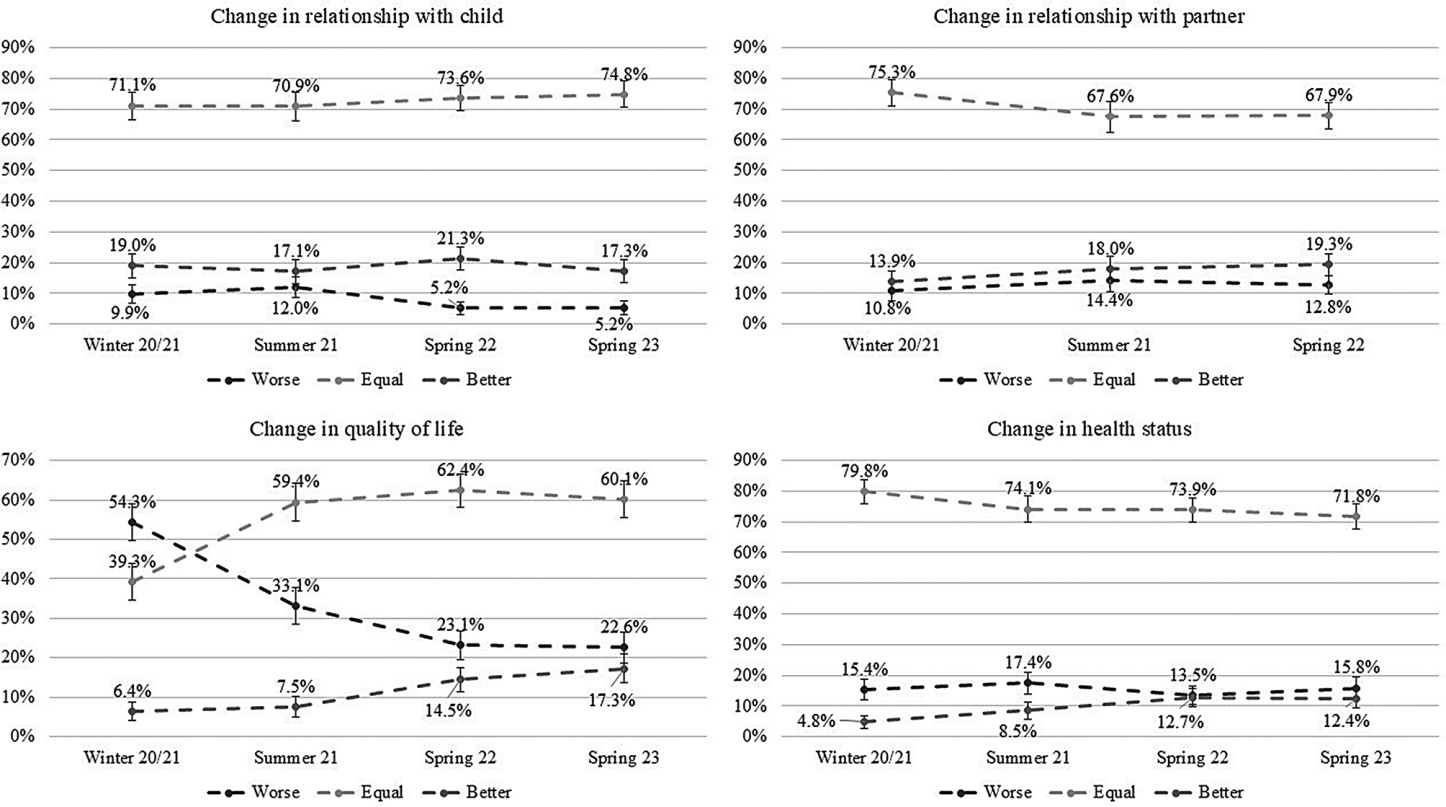
Percentages and 95% confidence intervals of the different groups of change in relationships over the different samples

Figure 1 displays the proportion of participants who evaluate their relationships with their child and partner, their quality of life and their health status as worse, equal, or better compared to before the pandemic across different stages of the pandemic. Approximately 75% of participants indicated that their relationship with their child remained unchanged throughout the course of the study. In winter 20/21 about 10% of participants rate their relationship as worse than it had been prior to the pandemic. The lowest rates were observed during spring 22 and spring 23 with about 5%, which marks a significant difference between summer 21 and spring 22. Concerning the relationship with the partner, in winter 20/21, three-quarters of participants evaluate their relationship with their partner as equal compared to before the pandemic, with no significant differences between the time points. In winter 20/21, about 10% of participants rate their relationship as worse than before the pandemic, with no significant differences between the time points. With regard to the quality of life in winter 20/21, about 40% of respondents indicated that their quality of life remained unchanged in comparison to before the pandemic. This increases continuously to about 60% in spring 23 with a significant difference between winter 20/21 compared to the other time points. In winter 20/21, about half of the participants rate their quality of life as worse compared to before the pandemic. This decreases throughout the samples, reaching approximately 23% with significant differences between winter 20/21 and summer 21 and between summer 21 and spring 22. Concerning changes in health status, more than three-quarters rate their health status as remaining unchanged during winter 20/21, without significant differences. About 15% indicated that their health status had deteriorated during the different time points, with only slight, non-significant changes.

### Change in relationships, quality of life and health status stratified by income

**Fig. 2:**
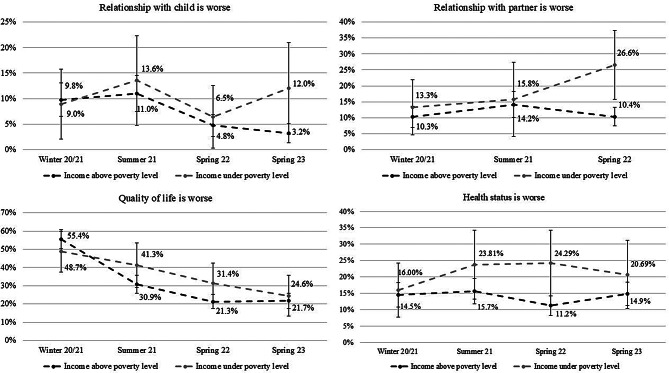
Distribution of percentages in dependence of income for the groups with worse relationships, quality of life and health status

Figure 2 displays the distribution in dependence of income below or above the poverty level of the groups which rate their relationship with the child and their partner, their quality of life and their health status as worse than before the pandemic. Concerning relationships with the child, the data indicate higher percentages in summer 21, with the highest percentage observed in the group below the poverty level However, the observed differences are not statisticallysignificant, with overlapping confidence intervals. Concerning the relationship with the partner, the group below the poverty level exhibits a strong increase. This indicates that, in the samples, constantly more people (proportionally) are in the group with a worse relationship with their partner. However, the confidence intervals between time points remain largely overlapping. But all in all, the group with income above the poverty level consistently exhibits lower percentages than the group below the poverty level, with even a significant difference in spring 23. Concerning the quality of life, there is a strong decrease in percentages in the group with an income above the poverty level with significant differences between winter 20/21 and summer 21, as well asbetween summer 21 and spring 22. In the group with an income below the poverty level, there was a decrease, but it was not as pronounced as in the group with an income above the poverty level. Concerning health status, the group with an income below the poverty level consistently exhibits higher percentages, though, no significant differences were observed between groups or time points.

### Change in relationships, quality of life, and health status stratified by gender

**Fig. 3:**
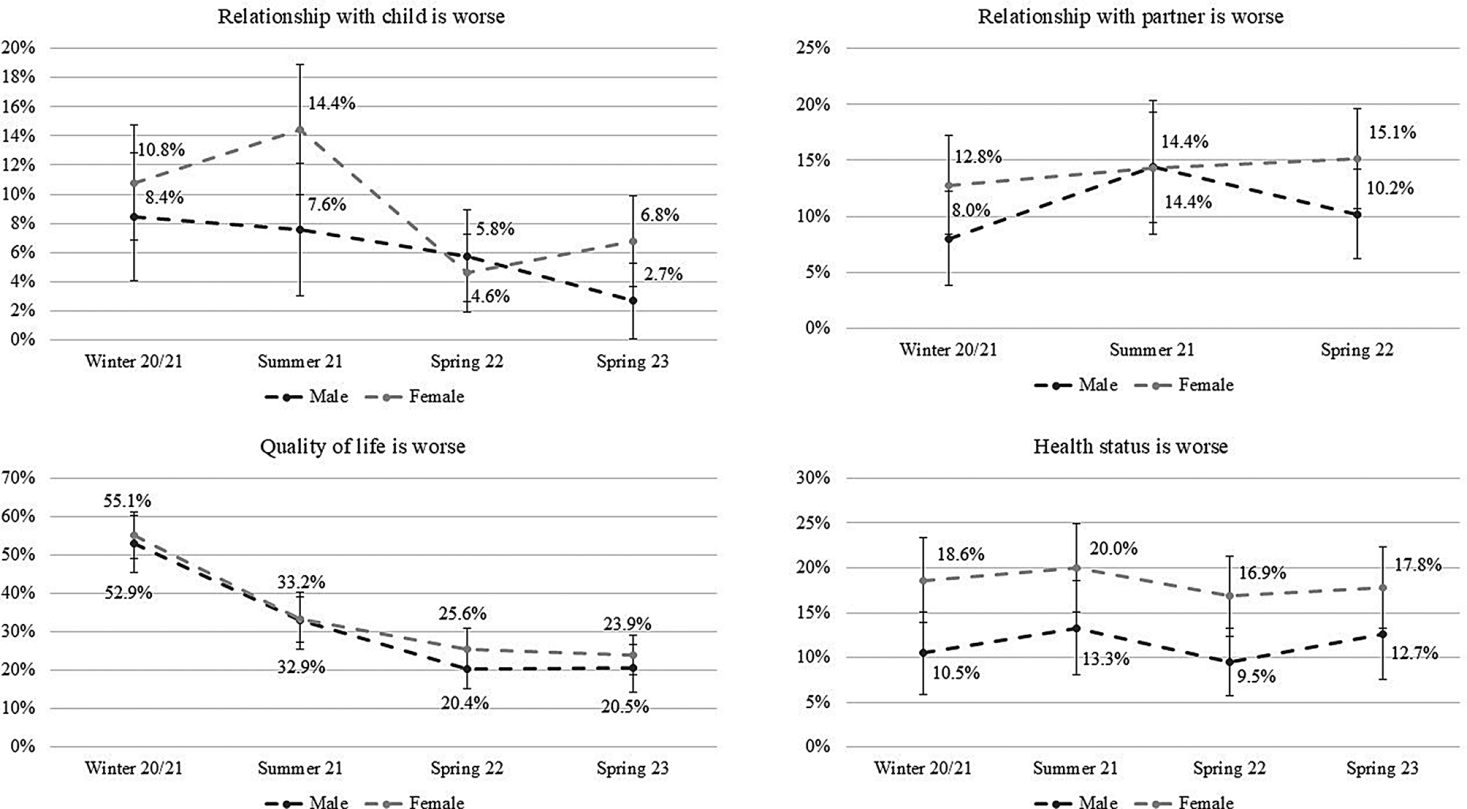
Distribution of percentages in dependence of gender for the group with a worse relationship with their child, their partner, a worse quality of life and health status

Focusing on gender differences, in the relationship with the child for females an initial increase, followed by a strong decline and a slight subsequent increase can be seen. The male cohort exhibited a slight decrease. Interestingly, during winter 20/21, summer 21, and spring 23, the percentage is lower for men, whereas during spring 22 females have a lower percentage. However, no significant differences were identified between the groups or time points, with the exception of the observed decrease in the group of females from summer 21 to spring 22. Concerning relationships with the partner, the percentage of males is constantly lower than the percentage of females, without significant differences between groups or time points. Quality of life increased strongly from about 55% to about 22%, for both males and females. The decreases observed from winter 20/21 to summer 21 were significant in both groups, However, the decrease between summer 21 and spring 22 was only significant in the male group. Concerning health status, similar patterns emerge for males and females across the various time points, but with constantly higher percentages for females. All in all there were no significant differences between groups or time points (see Fig. 3).

### Change in relationships, quality of life, and health status stratified by symptoms of depression and anxiety

**Fig. 4:**
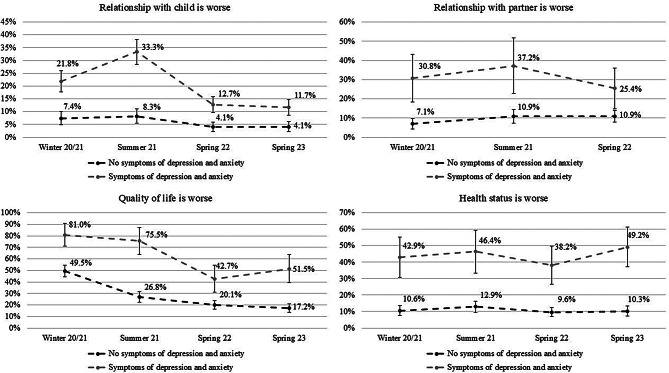
Distribution of percentages in dependence of symptoms of depression and anxiety for the groups with a worse relationship with their child, their partner and worse quality of life and health status

Figure 4 displays the distribution of our assessments of family functioning stratified by symptoms of depression and anxiety in the target parent, within the groups that rate their relationship with their child and their partner, as well as their quality of life, as being worse prior to the pandemic. Equally for relationships with the child, relationships with the partner and quality of life, we see constantly higher percentages in the group with symptoms of depression and anxiety throughout the samples compared to the group without symptoms of depression and anxiety. Concerning relationship with the child, the percentage in the group exhibiting symptoms of depression and anxiety increases significantly from winter 20/21 to summer 21, followed by a strong decrease in spring 22. The group without symptoms of depression and anxiety only exhibited slight non-significant changes. Importantly, the group differences are significant at every time point, with the exception of the period between spring 22 and spring 23. Concerning the relationship with the partner, we see no significant within-group differences between time points, although between-group differences were observed at each time point. With regard to quality of life, in the group with symptoms of anxiety and depression, a significant decrease from summer 21 to spring 22 was observed. In the group without symptoms of anxiety and depression, we find a significant decrease from winter 20/21 to summer 21. For quality of life, the between-group differences, but not the within-group differences, are significant at every time point. Concerning health status, it is notable that the percentage of respondents in the group with symptoms of anxiety and depression consistently exceeds that of the other group.

### Change in relationships, quality of life and health status stratified by the number of children

**Fig. 5:**
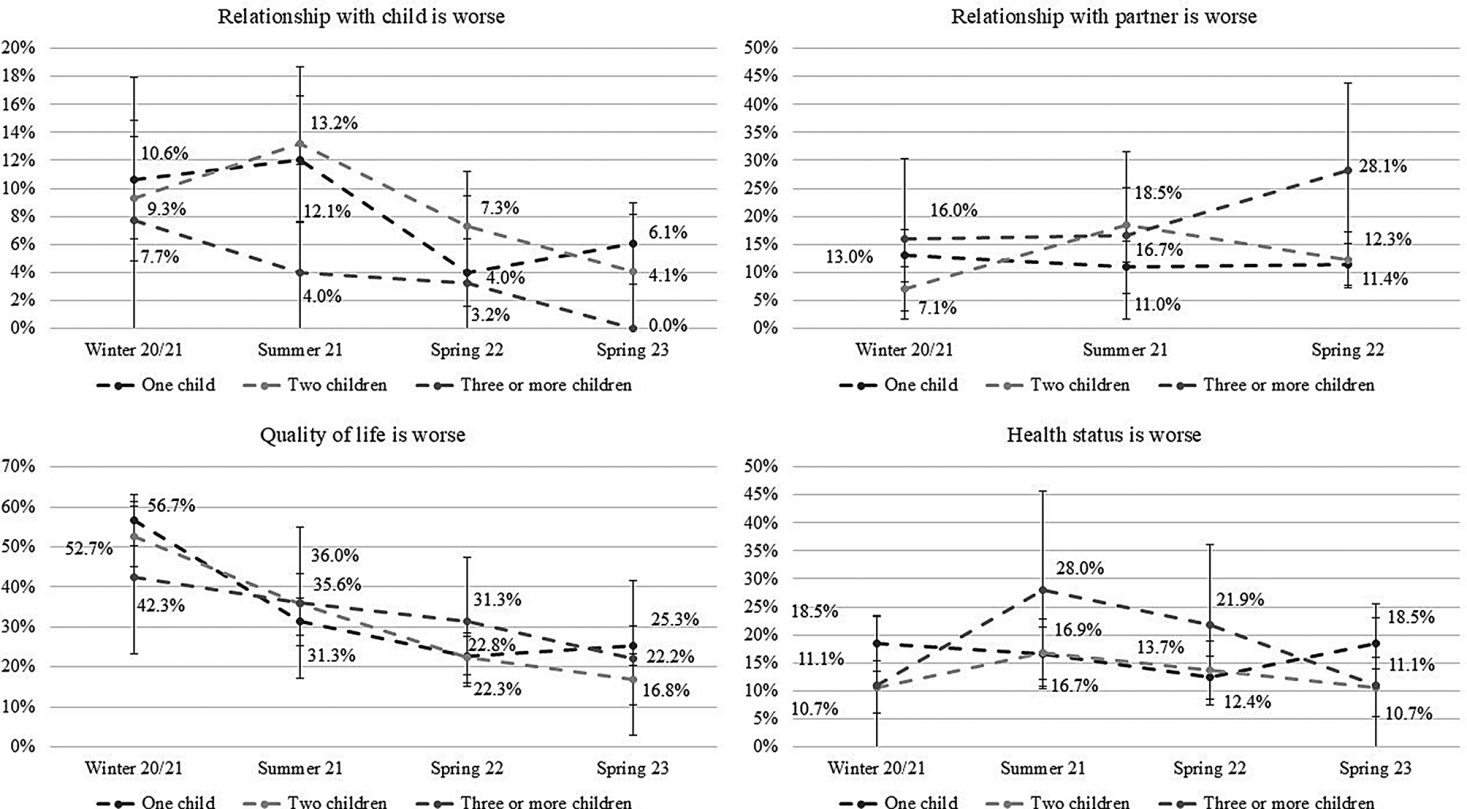
Distribution of percentages in dependence of number of children for the groups with a worse relationship with their child, their partner, worse quality of life and health status

All in all, an examination of the number of children in the relationship with the child reveals no between-group differences. For the group with three or more children, there is a constant decrease across all samples. In the case of the group with one child, a pronounced decline was observed during the spring 22, representing the sole significant in-group difference. Concerning relationship with the partner, all of the within-group and between-group differences are non-significant. With regard to quality of life, a constant decrease is observed across all samples for all groups, without significant between-group differences. However, a significant decrease was found from winter 20/21 to summer 21 for the groups with one child and two children. Concerning health status, the within-group changes across the time points, as well as the between-group differences are non-significant (see Fig. 5).

### Associations of the time points and covariates with worsening in relationship with child and partner, quality of life and health status

Associations were examined, using binary logistic regression analysis. Four models were calculated using relationship with child, relationship with partner, quality of life and health status as dependent variables. As independent variables the time points, symptoms of depression and anxiety, income, gender and the number of children were used. Being in the group which reports a worsening in the relationship with the child compared to before the pandemic was associated with symptoms of depression and anxiety (OR = 4.01, *p* < .001).Furthermore, significant differences were observed between summer 21 and spring 22 (OR = 0.42, *p* < .01). For detailed results see supplement Table 11. Being in the group which reports a worsening in the relationship with the partner was significantly associated with symptoms of anxiety and depression (OR = 4.12, *p* < .001), but without any other predictors or time differences being significant. For detailed results see supplement Table 12. Significant differences were observed in quality of life between winter 20/21 and summer 21 (OR = 0.40, *p* < .001) as well as between summer 21 and spring 22 (OR = 0.57, *p* < .001). Furthermore, being in the group which reports a worsening in quality of life was associated with symptoms of anxiety and depression (OR = 4.87, *p* < .001). For detailed results see supplement Table 13. Being in the group which reports a worsening in health status was associated with symptoms of anxiety and depression (OR = 5.84, *p* < .001) and gender (OR = 0.73, *p* = .04), but without significant differences between the time points. For detailed results see supplement Table 14.

## Discussion

In this study, we aimed to describe the changes in family functioning across four German population-based surveys during different stages of the COVID-19 pandemic between 2020 and 2023. Our study is, to the best of our knowledge, the first to examine relationships, quality of life and health status during different stages of the pandemic among individuals living with children, using large-scaled population-based samples in Germany and including data up to 2023 and the endemic stage.

Taken together the majority in our sample reported no negative changes in their relationship with their child, partner, quality of life and health status during the pandemic. However, a small number of individuals did report negative changes at different time points. Symptoms of depression and anxiety was found to be associated with reports of worsened family functioning andfinancial strain. Regarding gender, for the data indicate that female parents bear a greater burden, while there were no differences concerning the number of children.

In a first step, changes in the relationship with the child and the partner, quality of life and health status were assessed. The majority of participants reported that their relationship with their child did not change negatively during the pandemic. Nevertheless, there was a small group of participants with negative changes, which reduced significantly as the pandemic went on to about 5%. Similarly, a small group (13%) reported consistent negative changes in their relationship with their partner during the different stages. These groups represent small at-risk groups for further negative consequences. This is in line with previous research, indicating that 4% of parents experienced relationship difficulties as a stressor during the pandemic [16], as well as more conflicts [17]. Identifying this group is of vital importance, taking into account the association of heightened parental stress and child abuse [16], as well as good marriage quality being a protective factor against depression for mothers [18].

A notable decline in the quality of life of the participants was observed, with a considerable proportion of them rating their quality of life as worse. One potential explanation may be, that the strict measures were progressively relaxed throughout the different stages, of the pandemic, which may have contributed to this observed decrease. Interestingly in spring 23 about one-quarter reported that their quality of life had remained negatively affected compared to before the pandemic. This may be attributed to the loss of social networks or the permanent closure of activities due to the pandemic. These findings are consistent with previous research, indicating that initially high burdens are followed by decreases [33, 34]. These results are encouraging, as they indicate that the majority of the participants in our sample demonstrated resilience in the face of the pandemic, with no severe negative consequences. Conversely, the small at-risk-group seemed to exhibit ongoing negative changes, which warrant further investigation to ascertain the underlying causes of these declines in quality of life. Future studies should consider using a mixed methods approach to gain a more comprehensive understanding of the underlying factors influencing changes in family functioning during public health crises. Furthermore, this could facilitate the implementation of t appropriate support measures, to prevent severe long-term consequences.

Our findings regarding overall health status are encouraging, due to the result that about three-quarters of the parents rated their health status as equal to that which they experienced prior to the pandemic. Besides, we observed slight but significant improvements across the samples, although these increases were not robustly significant in the regression analysis. Consequently, the majority of parents in our sample did not experience a decline in their health status and demonstrated resilience during the pandemic. Nevertheless, a minority reported ongoing negative changes in their health status. This group should be examined further, to explore the reasons for the observed reduction in health status. One potential explanation for this reduced health status could be the presence of Long-Covid syndrome. Recent studies have indicated that about 10% of individuals experienced long-term symptoms for at least 12 weeks after infection [54]. Another reason could be the cessation of preventive measures during the acute phases of the pandemic. It would be beneficial to provide targeted health support for this group in the subsequent phase.

Together, our findings suggest that the majority of families in our sample coped well with the pandemic overall and were resilient in different domains of family functioning, as we saw that most participants were in the groups which reported no changes. Nevertheless, in each domain, there were small at-risk groups that exhibited persistent negative changes. These changes may be associated with the various long-term negative consequences for the risk-groups described above. Therefore, identifying and supporting these at risk-groups should be an important goal of social support systems.

The second objective was, to investigate differences in family functioning as a function of well-known risk factors, specifically low income, female gender, symptoms of anxiety and depression and having more children [13, 27–29].

A greater proportion of participants with an income below the poverty level reported worse relationships with their child and partner, with an increase observed in spring 22 and spring 23. However, these differences were only significant for the relationship with the partner in spring 23. This indicates that income may be a risk factor to long-term negative changes for the relationships in the family. One potential explanation could be, that financial strain constraints coincide for example the experience of reduced living space, because ogthe strict lockdown and curfew measures. This may have led to an increase in conflict within the family, as the limited space available provided fewer opportunities for each family member to retreat and engage in private activities. Interestingly, the disparity between the groups was highest during the last wave. Possible relevant factors may be the war in the Ukraine and the subsequent energy crisis. This is alarming and may be attributed to the prevailing challenging circumstances for families experiencing financial strain. This finding underscores the necessity for support for these at-risk families. Concerning quality of life, a similar pattern was observed for the two groups with a decrease, although this decrease was only significant for the group with an income above the poverty level. This is consistent with prior research indicating that financial strain is a risk factor for quality of life during [31] and before the pandemic [55]. Regarding the health status, we saw constantly higher percentages for parents with an income below the poverty level, although the observed difference was not-significant. This is unexpected, given that research prior to the pandemic, confirmed financial strain as a risk factor for deterioration in health [56]. Furthermore, child poverty is associated with with several long-term negative consequences, outlined in a review [57]. Surprisingly however, no evidence of an association between income and worsening in relationships, health status or quality of life was found in the regression analysis. This hints towards an interaction effect between time and income, with financial strain potentially acting as a moderating factor for lower family functioning over time in our sample. The above-described possible negative consequences strengthen the necessity for sufficient financial support of families in times of crisis.

Concerning gender, no differences were observed in relationships with the partner. Fewer women reported a worse relationship with their child during spring 22 compared to summer 21. The overall positive changes in quality of life reported above were consistently significant only for men. This finding aligns with those of several studies that have demonstrated a lower quality of life for females [29, 31, 58–60]. However, it contrasts with the results of our previous research conducted during an early stage of the pandemic [30]. Concerning relationships and health status, women tend to have slightly higher percentages. In our regression analysis, male gender was associated with a worsening in health status across all the samples. In sum, this hints towards a greater burden for women but should be further investigated in future research to confirm this initial finding.

Regarding quality of life, health status and the relationships with child and partner, the group with symptoms of depression and anxiety constantly demonstrated higher percentages, with these between-group differences being significant. Furthermore, symptoms of anxiety and depression were consistently associated with a worsening in health status, relationships and quality of life across all regression models. This is in line with previous research [29, 61] and has been confirmed before the pandemic as well [62]. For people with mental health symptoms higher stress levels have been confirmed [63]. This combined with the various stressors experienced during the pandemic, may explain for the higher percentages with worse relationships, health status and quality of life for people with symptoms of anxiety and depression. This confirms the need for special attention and support for people with mental health symptoms during the pandemic.

Differences in the number of children did not show any pattern in terms of quality of life, health status and the relationships with the child and partner. One explanation could be, that a growing number of children on the one hand can be a stressor with more caregivingand conflicts, but it can also have positive effects. For example, older siblings can help care for the younger ones and siblings can play together.

Overall, the quality of life in our sample was significantly lower at the beginning of the pandemic with remarkable increases until spring 2023. Similarly, the number of persons in our sample who reported a worsening in their relationship with their child during the pandemic has halved over the time points assessed. Symptoms of depression and anxiety were shown to be associated with declines in all domains of family functioning assessed in our sample. The relevance of having an income below the poverty level increased over the assessed time points and was significantly associated with a worsening of the relationship with the partner in spring 23. Number of children showed no consistent association with family functioning in our sample.

### Strengths and limitations

The main strength of this study is, that all samples were recruited in population-representative surveys with a consistent, high-quality methodology and reliable response rates (consistently > 40%). These response rates did not change during the pandemic, thus allowing the assessment of changes over time, overcoming the bias of dropout and selection bias of longitudinal samples and enabling valid monitoring of the medium-term consequences of the pandemic in the German population. Therefore, we can ensure a robust sample, by using systematic area sampling and assume high generalizability of the results. However, some limitations must be considered. First, although the main samples were population-based, only a subsample of them was used, which may limit the generalizability of results. Therefore, the generalizability is limited to parents/caregivers with a minor below the age of 16 years in the household. Furthermore, participants with non-sufficient language skills were excluded, resulting in the exclusion of many families with a migration background. Future research should specifically include this group, possibly with the use of translators. Second, participants were included if they lived in the same household with at least one person below the age of 16 years. Thus, we cannot exclude the possibility that non-parents were questioned. Only self-report measures were used, which may have been influenced by social desirability. However, the setting of questionnaires in sealable envelopes was chosen to reduce this effect and to ensure confidentiality. Furthermore, we investigated retrospective recalls of the changes at each time point. The self-report questions were not tested in a pilot study, due to time constraints, therefore the reliability and validity of these questions may be limited. Future research should use more objective and direct measures to investigate relationships and quality of life. In addition, seasonal effects on quality of life may limit the validity of our results. Furthermore, there could be a self-selection bias, as people who are willing to participate in the survey differ from people who refuse to participate. This could limit the validity of the results. Nevertheless, the sample was screened for representativeness at least in terms of sociodemographic characteristics, which may reduce this limitation. Furthermore, family functioning could be influenced by many more confounding variables like age, employment status or previous COVID-19 infections, which could not all be included in our survey. Nevertheless we tried to include the important factors with the stratifications, but of course with the stratifications raising the risk for bias due to multiple testing. Despite these limitations, our study provides important insights into trends in relationships and quality of life over different stages of the pandemic in large population-representative samples in Germany, using cross-sectional cohorts.

## Conclusion

Overall, negative changes in family functioning during the pandemic decreased continuously over the course of the pandemic in our sample. This is encouraging, as it indicates that, overall, families showed al resilient response and adjustment to the COVID-19 pandemic. Nevertheless, our results highlight an important at-risk group for ongoing negative consequences of the pandemic: the most prominent individuals with mental health symptoms or economic strain. Both findings underscore the necessity for psychosocial and therapeutic interventions targeting family functioning. For example, low-threshold support could be provided in schools, childcare institutions or at the pediatricians, during times of crisis. Furthermore, support systems need to be established to reduce the economic pressure on families in a non-bureaucratic way.

## Electronic supplementary material

Below is the link to the electronic supplementary material.

Supplementary Material 1

## Data Availability

The datasets used and/or analysed during the current study are available from the corresponding author on reasonable request.

## Abbreviations

RKI Robert: Koch-Institut
USUMA: Unabhängiger Service für Umfragen, Methoden und Analysen
ADM: Arbeitskreis Markt- und Sozialforschungsinstitute e.V.
PHQ-4: Patient health questionnaire 4
