# Supplementary material for: Trends in changes of family functioning during different phases of the pandemic – findings across four population-based surveys between 2020 to 2023 in Germany
Source: BMC Public Health. 2024 Nov 20;24:3230. doi: 10.1186/s12889-024-20650-2 (PMC11580525; doi:10.1186/s12889-024-20650-2)
Supplement: Supplementary file 1 — Supplementary Material 1 [file 12889_2024_20650_MOESM1_ESM.docx]

**Table 2**

*Main reasons for non-participation in the first study in winter 20/21.*

| **Reason for non-participation** | **non-participants in %** |
| --- | --- |
| refusal of the selected household to provide information | 23.8% |
| failure to contact persons in the household after four attempts | 14.1% |
| refusal of the target person to participate | 13.5% |

5,913 households were contacted; 2,519 took part in the survey; response rate: 42.6%

**Table 3**

*Main reasons for non-participation in the second study in summer 21.*

| **Reason for non-participation** | **non-participants in %** |
| --- | --- |
| refusal of the selected household to provide information | 24.0% |
| refusal of the target person to participate | 13.6% |
| failure to contact persons in the household after four attempts | 13.4% |

5,908 households were contacted; 2,515 took part in the survey; response rate: 42.6%

**Table 4**

*Main reasons for non-participation in the third study in spring 22.*

| **Reason for non-participation** | **non-participants in %** |
| --- | --- |
| refusal of the selected household to provide information | 23.4% |
| failure to contact persons in the household after four attempts | 13.9% |
| refusal of the target person to participate | 13.8% |

6,118 households were contacted; 2,522 took part in the survey; response rate: 41.2%

**Table 5**

*Main reasons for non-participation in the third study in spring 23.*

| **Reason for non-participation** | **non-participants in %** |
| --- | --- |
| refusal of the selected household to provide information | 24.3% |
| refusal of the target person to participate | 17.0% |
| failure to contact persons in the household after four attempts | 13.2% |

6,192 households were contacted; 2,515 took part in the survey; response rate: 41.3%

**Table 6**

*Change in relationship quality, quality of life and health status across different stages of the COVID-19 pandemic.*

| **Variable** | **Winter 20/21** | **Summer 21** | **Spring 22** | **Spring 23** |
| --- | --- | --- | --- | --- |
| **Change in relationship with child** |  |  |  |  |
| worse | 9.87%  (6.93–12.81) | 11.96%  (8.64–15.28) | 5.15%  (3.1-7.2) | 5.20%  (3.03-7.37) |
| equal | 71.14%  (66.67-75.61) | 70.92%  (66.28-75.56) | 73.60%  (69.51-77.69) | 74.80%  (70.55-79.05) |
| better | 18.99%  (15.12-22.86) | 17.12%  (13.27-20.97) | 21.25%  (17.46-25.04) | 17.30%  (13.60-21.00) |
| **Change in relationship with partner** |  |  |  |  |
| worse | 10.76%  (7.65-13.87) | 14.37%  (10.57-18.17) | 12.82%  (9.82-15.82) | - |
| equal | 75.33%  (71.00-79.66) | 67.59%  (62.52-72.66) | 67.85%  (63.65-72.05) | - |
| better | 13.91%  (10.44-17.38) | 18.04%  (13.87-22.21) | 19.33%  (15.78-22.88) | - |
| **Change in quality of life** |  |  |  |  |
| worse | 54.25%  (49.57-58.93) | 33.09%  (28.54-37.64) | 23.14%  (19.43-26.85) | 22.60%  (18.67-26.53) |
| equal | 39.31%  (34.72-43.90) | 59.37%  (54.62-64.12) | 62.37%  (58.11-66.63) | 60.10%  (55.49-64.71) |
| better | 6.44%  (4.13-8.75) | 7.54%  (4.99-10.09) | 14.49%  (11.40-17.58) | 17.30%  (13.74-20.86) |
| **Change in health status** |  |  |  |  |
| worse | 15.40%  (12.01- 8.79) | 17.43%  (13.77-21.09) | 13.45%  (10.45-16.45) | 15.83%  (12.40-19.26) |
| equal | 79.77%  (75.99-83.55) | 74.09%  (69.86-78.32) | 73.90%  (70.04-77.76) | 71.79%  (67.57-76.01) |
| better | 4.83%  (2.82-6.84) | 8.47%  (5.78-11.16) | 12.65%  (9.73-15.57) | 12.39%  (9.30-15.48) |

Percentages and 95% confidence intervals of the different groups of change in relationships over the different samples.

**Table 7**

*Change in relationships, quality of life and health status stratified by income.*

| **Variable** | **Winter 20/21** | **Summer 21** | **Spring 22** | **Spring 23** |
| --- | --- | --- | --- | --- |
| **Relationship with child is worse** |  |  |  |  |
| Income above poverty level | 9.81%  (6.53-13.09) | 11.00%  (7.46-14.54) | 4.75%  (2.61-6.89) | 3.21%  (1.34-5.08) |
| Income under poverty level | 8.96%  (2.12-15.80) | 13.56%  (4.82-22.30) | 6.45%  (0.34-12.56) | 12.00%  (2.99-21.01) |
| **Relationship with partner is worse** |  |  |  |  |
| Income above poverty level | 10.29%  (6.91-13.67) | 14.18%  (10.11-18.25) | 10.37%  (7.40-13.34) | - |
| Income under poverty level | 13.33%  (4.73-21.93) | 15.79%  (4.20-27.38) | 26.56%  (15.74-37.38) | - |
| **Quality of life is worse** |  |  |  |  |
| Income above poverty level | 55.39%  (50.13-60.65) | 30.86%  (25.93-35.79) | 21.29%  (17.37-25.21) | 21.68%  (17.48-25.88) |
| Income under poverty level | 48.68%  (37.44-59.92) | 41.27%  (29.11-53.43) | 31.43%  (20.55-42.31) | 24.56%  (13.39-35.73) |
| **Health status is worse** |  |  |  |  |
| Income above poverty level | 14.53%  (10.81-18.25) | 15.68%  (11.80-19.56) | 11.22%  (8.20-14.24) | 14.86%  (11.24-18.48) |
| Income under poverty level | 16.00%  (7.70-24.30) | 23.81%  (13.29-34.33) | 24.29%  (14.24-34.34) | 20.69%  (10.26-31.12) |

Percentages and 95% confidence intervals of the different groups of change in relationships over the different samples.

**Table 8**

*Change in relationships, quality of life, and health status stratified by gender*.

| **Variable** | **Winter 20/21** | **Summer 21** | **Spring 22** | **Spring 23** |
| --- | --- | --- | --- | --- |
| **Relationship with child is worse** |  |  |  |  |
| Male | 8.44%  (4.05-12.83) | 7.58%  (3.06-12.10) | 5.77%  (2.60-8.94) | 2.68%  (0.09-5.27) |
| Female | 10.79%  (6.87-14.71) | 14.41%  (9.93-18.89) | 4.60%  (1.94-7.26) | 6.75%  (3.65-9.85) |
| **Relationship with partner is worse** |  |  |  |  |
| Male | 8.02%  (3.84-12.20) | 14.39%  (8.40-20.38) | 10.22%  (6.26-14.18) | - |
| Female | 12.79%  (8.37-17.21) | 14.36%  (9.44-19.28) | 15.14%  (10.71-19.57) | - |
| **Quality of life is worse** |  |  |  |  |
| Male | 52.91%  (45.45-60.37) | 32.91%  (25.58-40.24) | 20.35%  (15.16-25.54) | 20.48%  (14.34-26.62) |
| Female | 55.13%  (49.12-61.14) | 33.20%  (27.40-39.00) | 25.56%  (20.32-30.80) | 23.88%  (18.78-28.98) |
| **Health status is worse** |  |  |  |  |
| Male | 10.47%  (5.89-15.05) | 13.29%  (8.00-18.58) | 9.52%  (5.74-13.30) | 12.65%  (7.59-17.71) |
| Female | 18.63%  (13.92-23.34) | 20.00%  (15.09-24.91) | 16.85%  (12.36-21.34) | 17.78%  (13.22-22.34) |

Percentages and 95% confidence intervals of the different groups of change in relationships over the different samples.

**Table 9**

*Change in relationships, quality of life, and health status stratified by symptoms of depression and anxiety.*

| **Variable** | **Winter 20/21** | **Summer 21** | **Spring 22** | **Spring 23** |
| --- | --- | --- | --- | --- |
| **Relationship with child is worse** |  |  |  |  |
| No symptoms of depression and anxiety | 7,44%  (4.84-10.04) | 8,28%  (5.46-11.10) | 4,09%  (2.25-5.93) | 4,11%  (2.17-6.05) |
| Symptoms of depression and anxiety | 21,82%  (17.73-25.91) | 33,33%  (28.51-38.15) | 12,73%  (9.64-15.82) | 11,67%  (8.53-14.81) |
| **Relationship with partner is worse** |  |  |  |  |
| No symptoms of depression and anxiety | 7.08%  (4.29-9.87) | 10.92%  (7.29-14.55) | 10.92%  (7.91-13.93) | - |
| Symptoms of depression and anxiety | 30.77%  (18.23-43.31) | 37.21%  (22.76-51.66) | 25.40%  (14.65-36.15) | - |
| **Quality of life is worse** |  |  |  |  |
| No symptoms of depression and anxiety | 49.46%  (44.35-54.57) | 26.82%  (22.23-31.41) | 20.09%  (16.29-23.89) | 17.20%  (13.37-21.03) |
| Symptoms of depression and anxiety | 80.95%  (71.25-90.65) | 75.47%  (63.89-87.05) | 42.65%  (30.90-54.40) | 51.51%  (39.45-63.57) |
| **Health status is worse** |  |  |  |  |
| No symptoms of depression and anxiety | 10.60%  (7.45-13.75) | 12.89%  (9.41- 6.37) | 9.56%  (6.78-12.34) | 10.30%  (7.20-13.40) |
| Symptoms of depression and anxiety | 42.86%  (30.64-55.08) | 46.43%  (33.37-59.49) | 38.24%  (26.69-49.79) | 49.21%  (37.15-61.27) |

Percentages and 95% confidence intervals of the different groups of change in relationships over the different samples.

**Table 10**

*Change in relationships, quality of life and health status stratified by the number of children*

| **Variable** | **Winter 20/21** | **Summer 21** | **Spring 22** | **Spring 23** |
| --- | --- | --- | --- | --- |
| **Relationship with child is worse** |  |  |  |  |
| One child | 10.63%  (6.43-14.83) | 12.06%  (7.54-16.58) | 3.98%  (1.56-6.40) | 6.08%  (3.19-8.97) |
| Two children | 9.26%  (4.80-13.72) | 13.19%  (7.66- 18.72) | 7.27%  (3.31-11.23) | 4.10%  (0.06-8.14) |
| Three or more children | 7.69%  (0-17.93) | 4.00%  (0-11.68) | 3.23%  (0-9.45) | 0% |
| **Relationship with partner is worse** |  |  |  |  |
| One child | 13.00%  (8.34-17.66) | 10.98%  (6.32-15.64) | 11.36%  (7.60-15.12) | - |
| Two children | 7.05%  (3.03-11.07) | 18.46%  (11.79-25.13) | 12.28%  (7.36-17.20) | - |
| Three or more children | 16.00%  (1.63-30.37) | 16.67%  (1.76-31.58) | 28.13%  (12.55-43.71) | - |
| **Quality of life is worse** |  |  |  |  |
| One child | 56.67%  (50.40-62.94) | 31.25%  (25.39-37.11) | 22.76%  (17.93-27.59) | 25.26%  (20.22-30.30) |
| Two children | 52.66%  (45.13-60.19) | 35.62%  (27.85-43.39) | 22.29%  (16.12-28.46) | 16.79%  (10.39-23.19) |
| Three or more children | 42.31%  (23.32-61.30) | 36.00%  (17.18-54.82) | 31.25%  (15.19-47.31) | 22.22%  (3.01-41.43) |
| **Health status is worse** |  |  |  |  |
| One child | 18.47%  (13.56-23.38) | 16.67%  (11.95-21.39) | 12.37%  (8.59-16.15) | 18.47%  (13.98-22.96) |
| Two children | 10.69%  (6.03-15.35) | 16.89%  (10.85-22.93) | 13.71%  (8.61-18.81) | 10.69%  (5.40-15.98) |
| Three or more children | 11.11%  (0-23.19) | 28.00%  (10.40-45.60) | 21.88%  (7.56-36.20) | 11.11%  (0-25.63) |

Percentages and 95% confidence intervals of the different groups of change in relationships over the different samples.

**Table 11**

*Association of differences in time points and the covariates with reporting a worse relationship with the child.*

| **Predictor** | **OR** | **OR 95%CI** | ***p*** |
| --- | --- | --- | --- |
| Constant | 0.08*** | - | <.001 |
| Difference between Winter 20/21 and Summer 21 | 1.29 | 0.79-2.09 | .31 |
| Difference between Summer 21 and Spring 22 | 0.42** | 0.24-0.72 | <.01 |
| Difference between Spring 22 and Spring 23 | 0.81 | 0.42-1.57 | .54 |
| Symptoms of depression and anxiety | 4.01*** | 2.60-6.17 | <.001 |
| Income under Poverty level | 1.11 | 0.67-1.84 | .68 |
| Male gender | 0.82 | 0.54-1.25 | .35 |
| Difference between one and two children | 1.24 | 0.83-1.85 | .30 |
| Difference between two and three children | 0.40 | 0.14-1.16 | .09 |

*Presented as Odds Ratio (OR). An OR > 1 corresponds to a higher probability of belonging to the group which reports a worsening in the relationship with their child during the pandemic with increasing values of the predictor; Nagelkerkes R^2^=.09; N=1,791.*

**Table 12**

*Association of differences in time points and the covariates with reporting a worse relationship with the partner.*

| **Predictor** | **OR** | **OR 95%CI** | ***p*** |
| --- | --- | --- | --- |
| Constant | 0.08*** | - | <.001 |
| Difference between Winter 20/21 and Summer 21 | 1.57 | 0.98-2.53 | .06 |
| Difference between Summer 21 and Spring 22 | 0.83 | 0.54-1.27 | .39 |
| Symptoms of depression and anxiety | 4.12*** | 2.73-6.21 | <.001 |
| Income under Poverty level | 1.54 | 0.97-2.46 | .07 |
| Male gender |  |  |  |
| Difference between one and two children | 1.00 | 0.68-1.47 | .99 |
| Difference between two and three children | 1.42 | 0.71-2.82 | .32 |

*Presented as Odds Ratio (OR). An OR > 1 corresponds to a higher probability of belonging to the group which reports a worsening in the relationship with their partner during the pandemic with increasing values of the predictor; Nagelkerkes R^2^=.09; N=1,791.*

**Table 13**

*Association of differences in time points and the covariates with reporting a worse quality of life.*

| **Predictor** | **OR** | **OR 95%CI** | ***p*** |
| --- | --- | --- | --- |
| Constant | 1.02 | - | .91 |
| Difference between Winter 20/21 and Summer 21 | 0.40*** | 0.30-0.54 | <.001 |
| Difference between Summer 21 and Spring 22 | 0.57*** | 0.42-0.78 | <.001 |
| Difference between Spring 22 and Spring 23 | 0.93 | 0.67-1.29 | .67 |
| Symptoms of depression and anxiety | 4.87*** | 3.59-6.62 | <.001 |
| Income under Poverty level | 0.97 | 0.72-1.32 | .85 |
| Male gender | 1.02 | 0.81-1.27 | .89 |
| Difference between one and two children | 0.91 | 0.73-1.15 |  |
| Difference between two and three children | 0.85 | 0.51-1.43 | .54 |

*Presented as Odds Ratio (OR). An OR > 1 corresponds to a higher probability of belonging to the group which reports a worsening in quality of life during the pandemic with increasing values of the predictor; Nagelkerkes R^2^=.18; N=1,791.*

**Table 14**

*Association of differences in time points and the covariates with reporting a worse health status.*

| **Predictor** | **OR** | **OR 95%CI** | ***p*** |
| --- | --- | --- | --- |
| Constant | 0.12*** | - | <.001 |
| Difference between Winter 20/21 and Summer 21 | 1.27 | 0.85-1.89 | .24 |
| Difference between Summer 21 and Spring 22 | 0.72 | 0.49-1.06 | .10 |
| Difference between Spring 22 and Spring 23 | 1.19 | 0.80-1.76 | .39 |
| Symptoms of depression and anxiety | 5.84*** | 4.28-7.98 | <.001 |
| Income under Poverty level | 1.17 | 0.82-1.68 | .39 |
| Male gender | 0.73* | 0.54-0.98 | .04 |
| Difference between one and two children | 0.96 | 0.71-1.30 | .81 |
| Difference between two and three children | 1.55 | 0.88-2.76 | .13 |

*Presented as Odds Ratio (OR). An OR > 1 corresponds to a higher probability of belonging to the group which reports a worsening in health status during the pandemic with increasing values of the predictor; Nagelkerkes R^2^=.14; N=1,791.*
